# Supplementary material for: TGF-β Type II Receptor Punt Suppresses Antimicrobial Peptide Expression and Influences Development in Tribolium castaneum
Source: Insects. 2023 Jun 2;14(6):515. doi: 10.3390/insects14060515 (PMC10299313; doi:10.3390/insects14060515)
Supplement: Supplementary file 1 [file insects-14-00515-s001.zip › insects-2372673-supplementary.pdf]

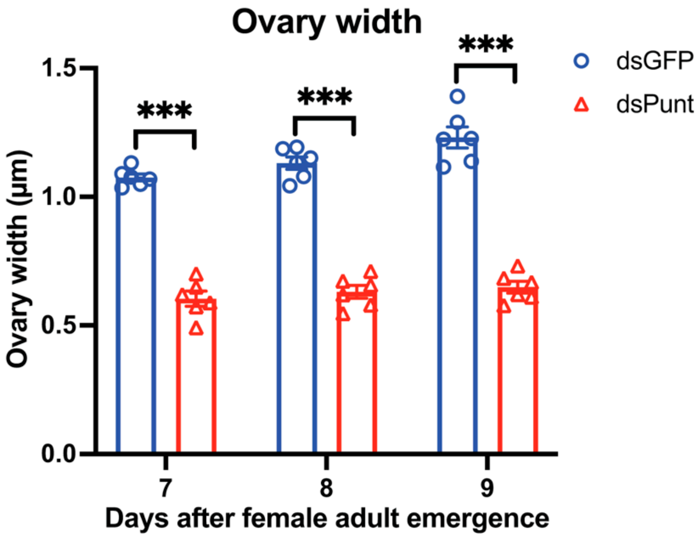
Ovary width
Ovary width (μm)
1.5
1.0
0.5
0.0
***
***
***
7
8
9
Days after female adult emergence
dsGFP
dsPunt

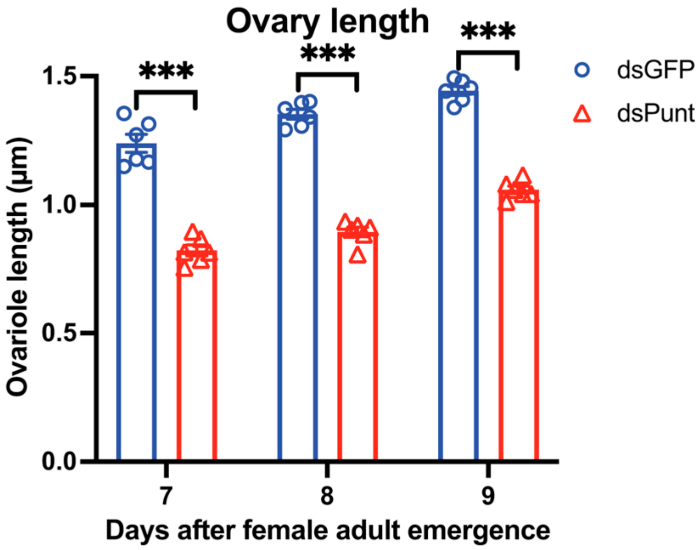
Ovary length
Ovariole length (μm)
1.5
1.0
0.5
0.0
***
***
***
7
8
9
Days after female adult emergence
dsGFP
dsPunt

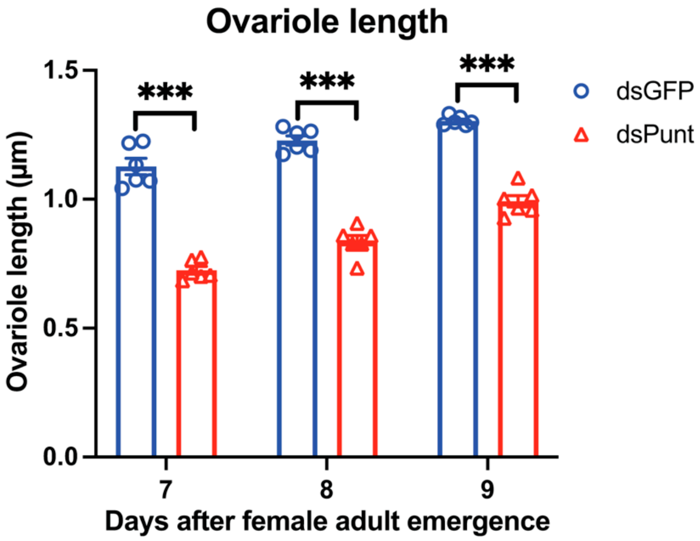
Ovariole length
Ovariole length (μm)
1.5
1.0
0.5
0.0
***
***
***
7
8
9
Days after female adult emergence
dsGFP
dsPunt

Figure S1. Ovarian width, length and ovariole length in dsGFP and dsPunt female adults. Asterisks above bars indicate significant differences between the treatment and corresponding control, * $p < 0.05$, ** $p < 0.01$, *** $p < 0.001$ by *t*-test.

Table S1. Primers used for dsRNA synthesis and qRT-PCR analysis

Primer name	Sequence (5′-3′)	Tm (℃)	Purpose
GFP-F	TAATACGACTCACTATAGGGTGGTCCCAATTCTCGTGGAAC	60.3	RNAi
GFP-R	TAATACGACTCACTATAGGGCTTGAAGTTGACCTTGATGCC	57.4	
Punt-F	TAATACGACTCACTATAGGGGTATCGCCTGAGCCAAATGT	58.3	
Punt-R	TAATACGACTCACTATAGGGTCCGACTTGTCGTAGCACTG	59.8	
Relish-F	TAATACGACTCACTATAGGGCTATCGGGCGATCACGTCAA	65.7	
Relish-R	TAATACGACTCACTATAGGGGGCCAAATTCTGCCACGTTT	65.7	
Tcrp3-F	TCAAATTGATCGGAGGTTTG	53.9	qRT-PCR
Tcrp3-R	GTCCCACGGCAACATAATCT	58.0	
Punt-F	AGCCTGTGCCGTGTCTTAGT	61.5	
Punt-R	TAAGTGTCCCGAACACCACA	58.9	
Relish-F	GAGCCTGTGCAGATTGATATG	53.5	
Relish-R	CTGGGGCAACATCGGTAAACA	57.8	
Att1-F	ATTTCCTCATGCCACTCGG	55.1	
Att1-R	CAAAAGCCCCCCCATCAAG	56.6	
Att2-F	CAAACGACCAAAGGGAAACTAAA	57.8	
Att2-R	TGAACTTGTCCAGTTGCATCGA	60.5	
Col1-F	TTGCCAGCCGAAGAGTACAA	56.7	
Col1-R	GTCTTTCTCGGTGGAGTTCA	54.3	
Def2-F	CCCTTTTCTGCATCTTCGAAAC	58.2	
Def2-R	CACATGCGGAATGGTTTAGCT	59.3	
Def3-F	TGCAATCACTGCTTACCCACTT	60.5	
Def3-R	ACAAGCAGCATGATTCACTTTGA	59.4	
